# Fine‐scale foraging habitat selection by two diving central place foragers in the Northeast Atlantic

**DOI:** 10.1002/ece3.7934

**Published:** 2021-08-24

**Authors:** Mathilde Huon, Yann Planque, Mark John Jessopp, Michelle Cronin, Florence Caurant, Cécile Vincent

**Affiliations:** ^1^ Centre d'Etudes Biologiques de Chizé UMR 7372 CNRS –La Rochelle Université La Rochelle France; ^2^ Observatoire Pelagis UMS 3462 CNRS ‐ La Rochelle Université, Pôle analytique La Rochelle France; ^3^ MaREI Centre Environmental Research Institute University College Cork Cork Ireland; ^4^ School of Biological, Earth & Environmental Sciences University College Cork Cork Ireland

**Keywords:** central place foragers, foraging activity, GPS/GSM telemetry, habitat selection, local scale

## Abstract

Habitat selection and spatial usage are important components of animal behavior influencing fitness and population dynamic. Understanding the animal–habitat relationship is crucial in ecology, particularly in developing strategies for wildlife management and conservation. As this relationship is governed by environmental features and intra‐ and interspecific interactions, habitat selection of a population may vary locally between its core and edges. This is particularly true for central place foragers such as gray and harbor seals, where, in the Northeast Atlantic, the availability of habitat and prey around colonies vary at local scale. Here, we study how foraging habitat selection may vary locally under the influence of physical habitat features. Using GPS/GSM tags deployed at different gray and harbor seals’ colonies, we investigated spatial patterns and foraging habitat selection by comparing trip characteristics and home‐range similarities and fitting *GAMMs* to seal foraging locations and environmental data. To highlight the importance of modeling habitat selection at local scale, we fitted individual models to colonies as well as a global model. The global model suffered from issues of homogenization, while colony models showed that foraging habitat selection differed markedly between regions for both species. Despite being capable of undertaking far‐ranging trips, both gray and harbor seals selected their foraging habitat depending on local availability, mainly based on distance from the last haul‐out and bathymetry. Distance from shore and tidal current also influenced habitat preferences. Results suggest that local conditions have a strong influence on population spatial ecology, highlighting the relevance of processes occurring at fine geographical scale consistent with management within regional units.

## Graphical abstract

Understanding animal‐habitat relationship at local scale is crucial in ecology, particularly if population are managed with regional units. This is the case of grey seals and harbour seals, whose trends in number vary among region in the Northeast Atlantic. The results suggest that local conditions might have a strong influence on their spatial ecology, highlighting the relevance of studying foraging habitat selection based on foraging behaviour at fine geographical scale.

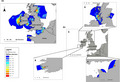

## 1 INTRODUCTION

Understanding species’ distribution and relationships with habitat is central in ecology and to the development of strategies for wildlife management and conservation (Morris, 2003; Rhodes et al., 2005). This is particularly true in marine ecosystems as oceans face increasing threats from overexploitation and habitat destruction (Halpern et al., 2008; Jones et al., 2018). In this ecosystem, distribution of a species is shaped by interactions between internal (i.e., species’ physiological tolerance, dispersal and reproductive strategies) and external factors (i.e., environmental features and regional richness). Depending on these different pressures, individuals disproportionately use the available conditions and resources, defining habitat selection (Mayor et al., 2009). This habitat selection differs from use or association as it implies choice and is commonly measured as use relative to availability or as use versus non‐use (Mayor et al., 2009). Habitat selection is an important component of animal behavior and a key determinant of individual survival, reproductive success, and ultimately population dynamic. Habitat selection can be defined at different scales (Johnson, 1980). Firstly, a coarse‐scale selection pertains the selection of species geographical range, determined by the dispersal and ability of species to relocate (Morris, 1992). At smaller (i.e., local) scale, habitat selection determines the use of various habitat characteristics of an individual or a social group within a home range. In the case of central place foragers, such as seabirds and pinnipeds, habitat selection at local scale is mainly performed around the colony. Then, in a metapopulation composed of different colonies in distinct geographical areas, habitat selection will vary locally due to the variation in physical habitat features and community structure. Furthermore, intra‐ and interspecific interactions might also drive spatial usage and consequently habitat selection through resource exploitation (i.e., prey depletion, Vance, 1984). The majority of studies on habitat selection of central place foragers focus on only one or two colonies within the metapopulation but few have compared the habitat selection across multiple colonies through a global analysis to establish a metapopulation‐level framework (e.g., Wakefield et al., 2017). However, understanding the causes of variation in habitat selection for local populations and determining how trends can be organized in space and time represents a major challenge in ecology (Fortin et al., 2008). Such an approach allows the development of coherent explanatory frameworks that carry across discrete populations and provide insights into phenotypic plasticity and eco‐evolutionary dynamics.

Selection of resources can be considered as the expression of different behavior forms (i.e., dispersal and migration) of an animal in a particular environment (Schoener, 1969). At a local scale, foraging behavior is perceived as the major behavior which ultimately influences reproductive success and survival rates (Breed et al., 2009; Morris, 1992) and was already taken into account in different studies focusing on habitat selection (Donazar et al., 1993; Duchamp et al., 2004; Monsarrat et al., 2013). Several studies on the ecology of marine central place foragers, such as pinnipeds and seabirds, used telemetry devices to incorporate foraging behavior in their analyses (Guinet et al., 1997; Hamer et al., 2001; Jonsen et al., 2007; Shiel et al., 1999).

In the Northeast Atlantic, gray seals (*Halichoerus grypus*) and harbor seals (*Phoca vitulina*) are two sympatric species occurring along the European continental, Irish and British coasts, with differing population trends (SCOS, 2017). Gray seal's and harbor seal's core populations are located in the UK with an estimated 141,000 and 43,500 seals, respectively (SCOS, 2017). In France and Ireland, colonies of both species are located at their southern and western limit of their European range, respectively. Seals move regularly between colonies and can remain at sea for a long period. However, they display a high degree of site fidelity and mainly forage around their haulout sites (Cronin et al., 2013; Huon et al., 2015; McConnell et al., 1999; Sjöberg & Ball, 2000). Both species are generalist and benthic feeders. Their diets vary regionally and seasonally (Breed et al., 2006; Hammond et al., 1994; McConnell et al., 1999; Spitz et al., 2010). Colonies of both species are located across a range of habitats and diet composition is likely related to prey availability and abundance surrounding the haulout region (Gosch et al., 2019).

This context represents an excellent case for comparing intra‐ and interspecific foraging habitat selection and spatial usage at local scale and to establish an interesting framework for seal species’ metapopulations. Many gray and harbor seals have been tracked from different colonies in the Northeast Atlantic, with analysis focusing on habitat selection from only one or two sites (Aarts et al., 2008; Bailey et al., 2014; Huon et al., 2015); or at the population scale (Jones et al., 2015) pooling multiple datasets. In this study, we combined for the first time several datasets for the assessment of foraging habitat selection across the wider population, but at local scale. Analyzing the data in this way enables new insights into gray and harbor seal's ecology across the Northeast Atlantic. We aimed at 1) studying the foraging habitat selection of gray and harbor seals at a colony (i.e., local) scale and 2) investigating the relationship between population trends and physical habitat features on the seals’ spatial patterns and foraging habitat selection.

## 2 MATERIALS AND METHODS

### 2.1 Study areas

**FIGURE 1:**
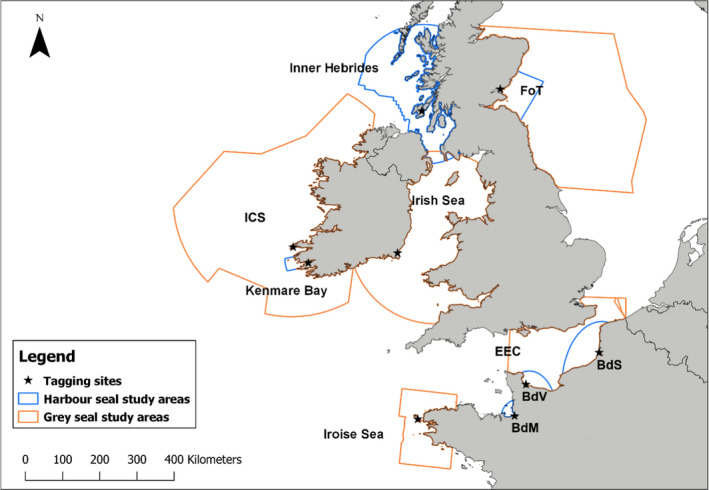
Map of the different study areas for gray seals (in orange): Irish Continental Shelf (ICS), Irish Sea, Firth of Tay (FoT), Iroise Sea, and Eastern English Channel (EEC); and harbor seals (in blue): Kenmare Bay, Inner Hebrides, Firth of Tay (FoT), Baie du Mont Saint michel (BdM), Baie des Veys (BdV), and Baie de Somme (BdS); including the tagging sites (black stars)

Gray seals were tracked in 5 regions (Figure 1, Appendix S1): the Irish Continental Shelf (*ICS;* seals were tagged on the Blasket Island), the Irish Sea (seals were tagged at Wexford), the Firth of Tay (*FoT*, representing the core population), the Eastern English Channel (EEC; seals tagged in the Baie de Somme, *BdS*), and the Iroise Sea (where gray seals are at the southern limit of their European range). Harbor seals were tracked in 4 regions: the Kenmare Bay, the Inner Hebrides, the Firth of Tay, and the English Channel, including the haulout sites of the *Baie du Mont Saint Michel (BdM)*, the *Baie des Veys (BdV)* and the *Baie de Somme*. Local seal numbers for each site was defined as follows: The raw number of individuals hauling out in each site during August (harbor seal molt, in 2012 for Irish sites, and 2013 for Scottish and French sites) was corrected with a conversion factor to consider the proportion of seals at sea during the surveys. These conversion factors were 0.72 and 0.24 (SCOS, 2020), respectively, for harbor seals and gray seals. When seals haul out continuously along the shore, a radius of 50 km was chosen around the capture site to count the raw number of seals. These calculations gave an estimate of local gray seal numbers of 488 seals for ICS, 996 in the Irish Sea (Duck & Morris, 2013), 2,008 in the FoT (Morris et al., 2021), 542 in the Iroise Sea, and 688 in the EEC (Vincent et al., 2017). Local harbor seals numbers were estimated at 476 in the Kenmare Bay (Duck & Morris, 2013), 4,411 in the Inner Hebrides, 69 in the FoT (Morris et al., 2021), 107 in the BdM, 152 in the BdV, and 629 in the BdS (Vincent et al., 2017) seals, respectively. Within all regions where both species are hauling out (Inner Hebrides, FoT, and EEC), we obtained tracking data from both species at FoT and EEC, providing the opportunity to study potential influence of interspecific interactions on foraging habitat selection and spatial usage.

### 2.2 Data description

#### 2.2.1 Seal handling and tagging

One hundred and two seals were caught and tagged in total (all tagging sites combined) between 2008 and 2014 by the University of La Rochelle (France), the Sea Mammal Research Unit (St Andrews University, UK), and University College Cork (Ireland), representing 46 gray seals and 56 harbor seals (Table 1). Seals were caught around and/or on their haulout sites after the molting period to optimize the tracking duration (as tags were glued on the fur), under licenses issued by the French ministry of the environment for France (Licenses Nos: 01/161/AUT, 01/525/AUT, 03/380/AUT, 05/475/AUT, 05/485/AUT, 06/82/AUT, 07/481/AUT, 08/346/DEROG, 08/347/DEROG, 10/102/DEROG, 11/873/DEROG,11/874/DEROG, and 13/422/DEROG.); by the National Parks & Wildlife Service (License Nos: C35/2008, C014/2012, C0019/2011, C04/C023/2013, and C016/2014), the Irish Health Products Regulatory Authority for Ireland (Project License AE19130/P004); license provided by UK Home (Licenses Nos: #60/2589, #60/3303 and #60/4009) in accordance with the Animals Scientific Procedures Act 1986 and the Scottish Government under the Conservation of Seals Act (1970) and the Marine (Scotland) Act (2010), and under license from Marine Scotland. Seals were fitted with Fastloc™ GPS/GSM tags developed by the Sea Mammal Research Unit that included a wet/dry sensor to determine whether the seal was hauled out, swimming at the surface, or diving. Haulout events started when the sensor was continuously dry for more than 10 min and ended when it was continuously wet for 40 s. GPS locations were attempted every 20 min resulting in irregular location intervals when seals were underwater or in areas of poor satellite coverage. Diving was recorded when a seal reached a depth greater than 1.5 m, with start and end time of dive, maximum depth, dive duration, and nine intermediate dive points recorded for each dive. The location of each dive was spatially interpolated from the true GPS locations. The recorded data were relayed through onboard mobile phone with GSM modem (Sea Mammal Research Unit, St Andrews University^1^). Some of these data were already used in previous publications (Table 1).

**TABLE 1:** Details of gray and harbor seals fitted with GPS/GSM tags in the different study areas: Iroise Sea, Eastern English Channel (EEC), Irish Sea; Irish Continental Shelf (ICS), Firth of Tay (FoT) for gray seals; Baie du Mont Saint Michel (BdM), Baie des Veys (BdV), Baie du Somme (BdS), Kenmare Bay, Inner Hebrides, and FoT

Species	Country	Catching area	Number of Seals	Number of males	Number of females	Body Mass (Kg ± *SD*)	Body length (cm ± *SD*)	Tracking duration (days ± *SD*)	Number of filtered points	Number of dive points	Number of foraging dive points	Publication
Gray seal	France	Iroise Sea	10	8	2	120 ± 54	166 ± 24	190 ± 29	62,092	303,910	71,588	Huon et al. (2015)
	France	EEC	8	8	0	135 ± 34	172 ± 20	178 ± 50	24,714	289,435	61,101	Planque et al. (2020)
	Ireland	Irish Sea	8	5	3	132 ± 36	177 ± 18	122 ± 84	28,770	179,550	73,514	Cronin et al. (2016)
	Ireland	ICS	10	2	8	104 ± 29	156 ± 12	192 ± 77	26,627	359,845	119,948	Gosch et al. (2014)
	Scotland	FoT	9	4	5	115 ± 24	171 ± 11	191 ± 66	17,239	300,084	112,163	Jones et al. (2015)
Harbor Seal	France	BdM	6	3	3	76 ± 19	135 ± 12	95 ± 45	13,048	117,910	19,408	Vincent et al. (2010)
	France	BdV	12	9	3	71 ± 9	133 ± 11	138 ± 40	25,765	182,074	46,225	Vincent et al. (2010)
	France	BdS	10	9	1	81 ± 11	142 ± 6	134 ± 53	13,201	388,368	62,145	Planque et al. (2020)
	Ireland	Kenmare bay	10	7	3	72 ± 11	139 ± 11	99 ± 48	11,269	276,247	78,170	Cronin et al. (2009)
	Scotland	Inner Hebrides	10	4	6	78 ± 11	143 ± 5	136 ± 121	45,014	202,700	57,707	Jones et al. (2015)
	Scotland	FoT	8	6	2	87 ± 14	144 ± 7	106 ± 43	39,305	224,982	54,329	Jones et al. (2015)

#### 2.2.2 Breeding and molting period

During the breeding and molting periods, seals tend to strongly reduce their foraging activities, increasing the amount of time spent hauled out, or staying close to their haulout sites (Boness, 1984; Caudron et al., 2009; Lidgard et al., 2003). We aimed to focus on foraging activities outside these periods when seals need to replenish their body reserves and are less constrained to haulout sites. Data obtained during the breeding (September to December for gray seals, June to July for harbor seals) and early molting (January to February for gray seals and August to September for harbor seals) periods were excluded from the analyses.

#### 2.2.3 Return‐trip selection

In the Northeast Atlantic, harbor seals undertake short movements from their haulout sites (10–20 Km) and show long‐term site fidelity (Ries et al., 1997; Tollit et al., 1998; Vincent et al., 2010). In contrast, telemetry on gray seals showed frequent movements between colonies (McConnell et al., 1999). They can alternate return trips to their haulout site in specific areas (within areas where most of their foraging activities occur) but they also frequently travel over hundreds of kilometers to distinct haulout site (SCOS, 2017). To study habitat selection and spatial usage of gray seals, we only selected individuals’ foraging return trips around the tagging colony (i.e., defined as haulout sites grouping), excluding travel trips and return trips from other areas (McConnell et al., 1999).

#### 2.2.4 Explanatory variables

Based on seals’ benthic foraging behavior (Bjørge et al., 1995; Hindell et al., 1991; LeBoeuf et al., 1988; Thompson et al., 1991), we used three environmental variables to identify habitat (Appendix S2). Bathymetry was obtained from the European Marine Observation and Data Network (*EMODnet*), with a grid size resolution of 0.125 × 0.125 min.^2^ Sediment data were obtained from the *MESH_EUNIS* model (Mapping European Seabed Habitat project), which predicts habitat types with a spatial resolution of 300 m. Sediment types were based on a simplified FOLK classification system (Folk, 1954) and limited to the most dominant types: rock, mud, sand, gravel, coarse, and mixed sediments. We used different tidal current datasets for Irish, Scottish, and French areas, scaled to similar resolutions for inter‐site comparison. Datasets for the French study areas were obtained from *Previmer* (Lecornu & De Roeck, 2009) for the tracking period. These were created from the MARS 2D model with a resolution of 250m and were available at an hourly scale. The Irish Marine Institute provided tidal current data for the Irish Continental Shelf and the Irish Sea^3^. Data were obtained from a numerical model with a spatial resolution varying between 1.2 Km and 1.5 Km and corresponded to surface tidal current at 3 hr interval. This dataset did not cover the Kenmare bay (for which no tidal current data were available). We averaged model datasets for the French and Irish areas, respectively, in order to represent the tidal strength in space irrespective of instant tidal phases (ebb, slack, or rising tide). Tidal current data for Scottish sites were obtained from the Web vision renewable website and were calculated from the ABP mer model (*Atlas of UK marine Renewable Energy Resources 2008* ^4^ *)*. These data corresponded to the peak current speed of a mean spring tide (m.s^‐1^), with a spatial resolution decreasing from 200 m to 5 Km from inshore to offshore areas.

The distance between each GPS location and the last haulout and the distance to the shore were also included as explanatory variables to describe accessibility to the environment (Aarts et al., 2008). The geodesic distance to the last haulout visited was calculated using the *LC.dist* function from the *Marmap* package (Pante & Simon‐Bouhet, 2013) in R *v 3.3.3* (R core Team 2017). Distance from shore was calculated as the straight‐line distance to the closest point along the coast using ArcGIS *v 10.5* (Environmental Systems Research Institute, Inc., Redlands, CA, 2017) “Nearest” function.

### 2.3 Foraging habitat selection

#### 2.3.1 Identification of seals foraging dives

We identified foraging behavior using a vertical approach based on two diving criteria: the dive shape and vertical descent speed (Planque et al., 2020). First, we excluded dives with a maximum depth <3 m and a dive duration <30 s, considering that these very shallow and short dives are unlikely to be foraging dives. The two dive criteria indicating benthic foraging were determined at the individual level to consider potential inter‐individual variability in diving due to physiological conditions, swimming capacities, and individual strategies (Austin et al., 2006; Beck et al., 2003), as suggested by Planque et al. (2020). Dive‐shape was determined using the *Time Allocation at Depth* index (Fedak et al., 2001). TAD values vary from 0 to 1, where 0 correspond to dives close to the surface (i.e., when the animal spent most of its dive time at a shallower depth than the maximum depth) and 1 represents “U‐shape” dives (i.e., when the animal spent most of its dive time at the maximum depth). Harbor and gray seals are generally considered benthic feeders and therefore mostly perform U‐shaped dives when they forage. Following Planque et al. (2020), we selected the most U‐shaped dives for each individual by selecting 25% of the highest TAD values. Because U‐shaped dives with a very low vertical descent speed are more likely associated with resting/sleeping behavior, we excluded a further 10% of the most U‐shaped with the lowest vertical descent speeds for each individual. We therefore selected ~22.5% of all dives that are more likely associated with foraging, and we used the interpolated location of these dives in habitat selection models.

#### 2.3.2 Use‐availability design

Following the use‐availability design (Keating & Cherry, 2004), we assessed the foraging habitat selection by comparing the environmental characteristics of foraging dive locations to those of randomly generated points (i.e., pseudo‐absences), representing the habitat availability (Aarts et al., 2008; Johnson et al., 2006; Keating & Cherry, 2004; Lele & Keim, 2006). Two pseudo‐absences per foraging dive point were created locally for each individual seal within the different study areas using the package *sp* in R. These random points were created in each study area within buffers three times the size of local population Minimum Convex Polygon (Burgman & Fox, 2003) of all seals’ dive locations, limited by the continental shelf (seals do not travel further offshore). For the *EEC* and the *FoT*, where both species were present, one buffer was created for each species.

#### 2.3.3 Modeling analyses

We fitted *generalized additive mixed models* (GAMM) to the data, with the *gam* function *mgcv* R package. We used a binomial family argument with a *logit‐link* function to estimate the parameters of an *inverse‐logit* selection model based on seal foraging dives and random points (Johnson et al., 2006). Foraging dives and pseudo‐absences were the response variable, taking the values 1 and 0, respectively. To consider intra‐individual autocorrelation, we included individual as a random effect. Environmental variables were treated as fixed effects. Bathymetry, tidal current, distance from shore, and distance from the last haulout were included as continuous variables; sediments were treated as categorical variable. When one sediment type was over‐represented, the model was forced to consider this sediment type as reference level (otherwise reference sediment type was included alphabetically). The multicollinearity between covariates was assessed using the VIF value (Kutner et al., 2004). The best model was selected using the AIC (Akaike, 1973). Firstly, we fitted one model per site for each species to focus at the local scale. Secondly, to highlight the importance of modeling habitat selection around colonies, particularly when local habitat characteristics differ, we also fitted a global model for each species using pooled data from all colonies and included “site” as a random factor. We did not include Kenmare for harbor seals’ global model, as tidal current dataset was not available for this area. However, as we had problem of convergence when running global model, we only included one diving point on three. For models fitted for each colony, we calculated the importance of each covariate using the prediction function of the GAMM, providing an index of the relative importance of each covariate in the chosen model. Maps of habitat selection predicted by the model were created with ArcGIS for all sites.

### 2.4 Influence of intra‐ and interspecific interactions on spatial patterns and home‐range segregation

Trip characteristics and measures of similarity between home ranges were used to evaluate the influence of intra‐ and interspecific interactions on spatial patterns, to get complementary information on foraging habitat selection. For each species, trips with duration lower than 3 hr were removed as they were considered to be in the vicinity of haulout sites (Cronin et al., 2013). We used trip duration and maximum distance from the haulout sites (values were log‐transformed to correct for non‐normal distribution). Interpolated tracks were used for these trip characteristics. To reduce sampling bias (between areas where seals spend more time diving or out of the water), we interpolated all GPS locations every 20 min using straight‐line interpolation. We assumed that each trip made by an individual seal was independent from the others. Shapiro and Bartlett tests were firstly used to test the normality and homoscedasticity of the data by using the functions *shapiro.test* and *bartlett.test* from the package *Stat* in R. If the normality and homoscedasticity were validated, ANOVA was used for comparing means of trip characteristics between the different sites; if not, we used a Kruskal–Wallis test (respectively *aov* and *kruskal.test* function). When the intervariability was validated, a post hoc test was used for pairwise comparison. We used a Tukey HSD test (*TukeyHSD* function) in the case of ANOVA; and the dunn test (*dunn.test* function in the *dunn.test* R package) in the case of Kruskal–Wallis. In addition, to test if the number of seals in the colony (i.e., density dependence) influence the spatial patterns, we fitted a linear effect model for each metric and species using the *lme* function of the *nlme* R package. Trip duration and maximum extent were used as response variable and were log‐transformed, number of seals at the colony and latitude were used as explanatory and continuous variables, and site was included as a random factor. The best model was selected using AIC criteria. We used the *Bhattacharyya's affinity* index (BA; Bhattacharyya, 1943) to quantify spatial overlap in home range. This method quantifies the spatial overlap between two population spatial distribution (Fieberg et al., 2005) and provides a value ranging from 0 (i.e., no overlap or complete segregation) to 1 (i.e., complete overlap). We applied the BA on the 95% Kernel density of foraging dive locations between individuals of the same colony to study the influence of colony size (i.e., indirectly the density dependence), and between species when both species were tracked around the same colony (i.e., FoT and EEC) to study the influence of interspecific interactions. We used the *Kerneloverlaphr* function of the package *adehabitatHR* in R (Calenge, 2006; Fieberg, 2014).

## 3 RESULTS

### 3.1 Foraging habitat selection

Only results obtained for models fitted at local scale are presented in this section. Global models highlighted an influence of colony in the foraging habitat selection (*p* < .05). Although overall the results obtained by the two types of models were mostly similar, some ecological inconsistencies were observed (i.e., positive influence of bathymetry for harbor seals in French colonies or distance from shore for gray seals in the EEC, in the predictions of foraging habitat selection, Appendices S4 and S5), suggesting a homogenization issue in the models.

#### 3.1.1 Gray seal foraging habitat selection

**FIGURE 2:**
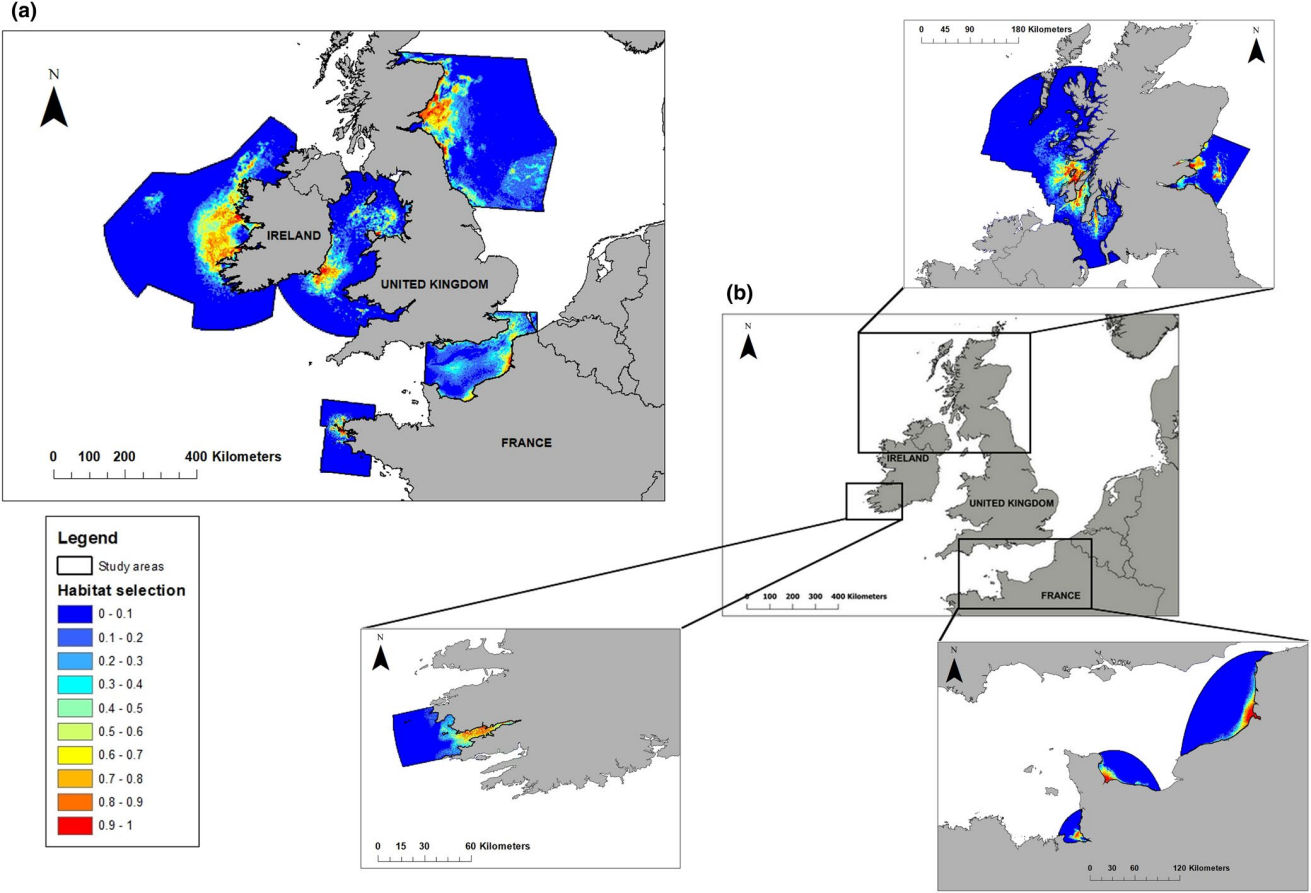
Habitat selection of gray seal for all study areas (a, top right); and of harbor seals (b, left) with magnified maps for the Inner Hebrides and the Firth of Tay (top); Kenmare bay (bottom left), and the Baie du Mont Saint Michel Baie des Veys and Baie de Somme (bottom right)

438,314 dive points were identified as foraging dive locations across all study areas (Table 1, Appendix S3). The details of the model selected for each site are presented (Table 2, Appendix S6). The explained deviances for all sites were relatively high (Table 2), varying between 31% (for the EEC) and 78% (for the Iroise Sea). For most sites, the distance from the last haulout accounted for most of the explained variance, varying from 45% (FoT) to 76% (Iroise Sea). The second variable having a strong influence on the habitat selection was the bathymetry, varying from 15% (Iroise Sea) to 40% (FoT) of the explained deviance. These two parameters had a negative influence on foraging habitat selection; gray seals tended to select foraging habitat close to their haulout sites and in shallower waters. Distance from shore explained 10% or more in some of the study areas. Tidal current and sediments combined accounted for less than 10% of the explained deviance, but the influence of these variables differed among sites. In the EEC and ICS, gray seals selected habitat further than 20 Km and 200 Km from shore, respectively. Conversely, gray seals selected their foraging habitat less than 50 Km from shore in the Irish Sea. Tidal current speed had a positive influence on gray seals’ foraging habitat selection in the Irish Sea. For the other sites, seals selected a minimum value of tidal current speed (1.5 and 0.4 m/s for the Iroise Sea and the FoT, and 0.6 m/s for the EEC). Gray seals selected different types of sediments in the different study areas. Habitat close to the colonies was highly selected in all study areas (Figure 2). This was particularly true for the Iroise Sea and the Irish Sea, where gray seals mainly selected their foraging habitat in shallow waters around tidal areas, where they haul out.

**TABLE 2:**
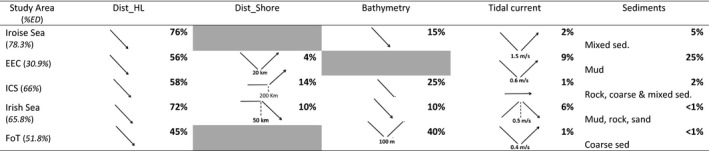
Influence of environmental characteristics on gray seal's habitat selection and explained deviance in percentage (%ED) for each study area

Note

For each environmental variable the percentage of the explained deviance is showed (in bold). Gray boxes indicate variables dropped by the model selection.

#### 3.1.2 Harbor seal foraging habitat selection

359,001 dive points were identified as foraging dive locations across all study area (Table 1, Appendix S3). Details of the models selected for each site are presented in Table 3. The overall explained deviance (ED) was relatively high (Table 3, Appendix S7) varying from 30.9% (BdV) to 78.3% (BdM). Distance from the last haulout (91% of ED for the Inner Hebrides), the distance from shore (92% of ED for the BdS), and the bathymetry (62% of ED for the BdV) predominantly explained the deviances. The distance from the last haulout had a negative influence for all sites, that is, harbor seals selected their foraging habitat close to their haulout sites. The influence of distance from shore and bathymetry were more contrasted. For BdS and Kenmare bay, harbor seals selected short distances from shore. Nevertheless, in the Inner Hebrides and FoT, harbor seals selected their foraging habitat at 20 and 40 Km from the coast, respectively. Harbor seals selected habitat in shallow waters in the BdM and the BdV. In the Inner Hebrides and the FoT, they selected depths at 40 and 25 m, respectively. Tidal current and sediment together only explained less than 10% of the deviance, except for the Firth of Tay where tidal current explained 55% of the deviance. Habitat selection was highest along the coastline for the BdS, and within the bays for the BdM, the BdV and Kenmare bay (Figure 2). In the Inner Hebrides and the FoT, harbor seals selected their foraging habitat in inshore and in distance to the shore.

**TABLE 3:**
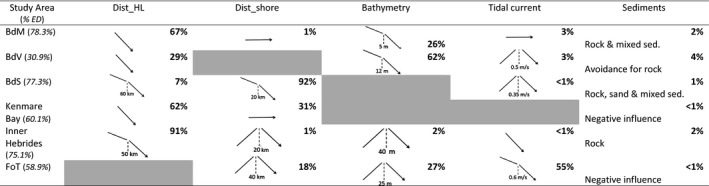
Influence of environmental characteristics on harbor seal's habitat selection and explained deviance in percentage (%ED) for each study area

Note

For each environmental variable the percentage of the explained deviance is showed (in bold). Gray boxes indicate variables dropped by the model selection.

### 3.2 Influence of intra‐ and interspecific interactions on spatial usage and home‐range segregation

For each site and species, the hypotheses of normality and homoscedasticity of trip duration and maximum extent were rejected (*p* < .05) leading to the use of the nonparametric Kruskal–Wallis test and Dunn test as a post hoc test. For both metrics and both species, the model containing only latitude was selected (Table 4) and its influence was not significant in all cases. Seal numbers at the colony did not influence the spatial pattern of each species.

**TABLE 4:** The 3 candidate models of the two spatial pattern metrics: trip duration (log_trip_duration); maximum extent (log_maximum_extent), with Akaike's information criterion (AIC) values

	AIC (gray seals)	AIC (harbor seals)
*Trip duration*		
Log_trip_duration ~number of seals + re(sites)	12,909	12,189
**Log_trip_duration ~latitude + re(sites)**	**12,898 *p* > .05**	**12,179 *p* > .05**
Log_trip_duration ~number of seals + latitude + re(sites)	12,914	12,194
*Maximum extent*		
Log_maximum_extent ~number of seals + re (sites)	13,577	12,098
**Log_maximum_extent ~latitude + re (sites)**	**13,566 *p* > .05**	**12,085 *p* > .05**
Log_maximum_extent ~number of seals + latitude + re (sites)	13,577	12,106

Note

Model selected for each metric, with the lowest AIC, is in bold. *p* < .05 is the value of latitude variable in the model. The 3 candidate models of the two spatial pattern metrics: trip duration (log_trip_duration); maximum extent (log_maximum_extent), with Akaike's information criterion (AIC) values. Models selected with the lowest AIC value are in bold.

#### 3.2.1 Gray seals’ trip characteristics

**FIGURE 3:**
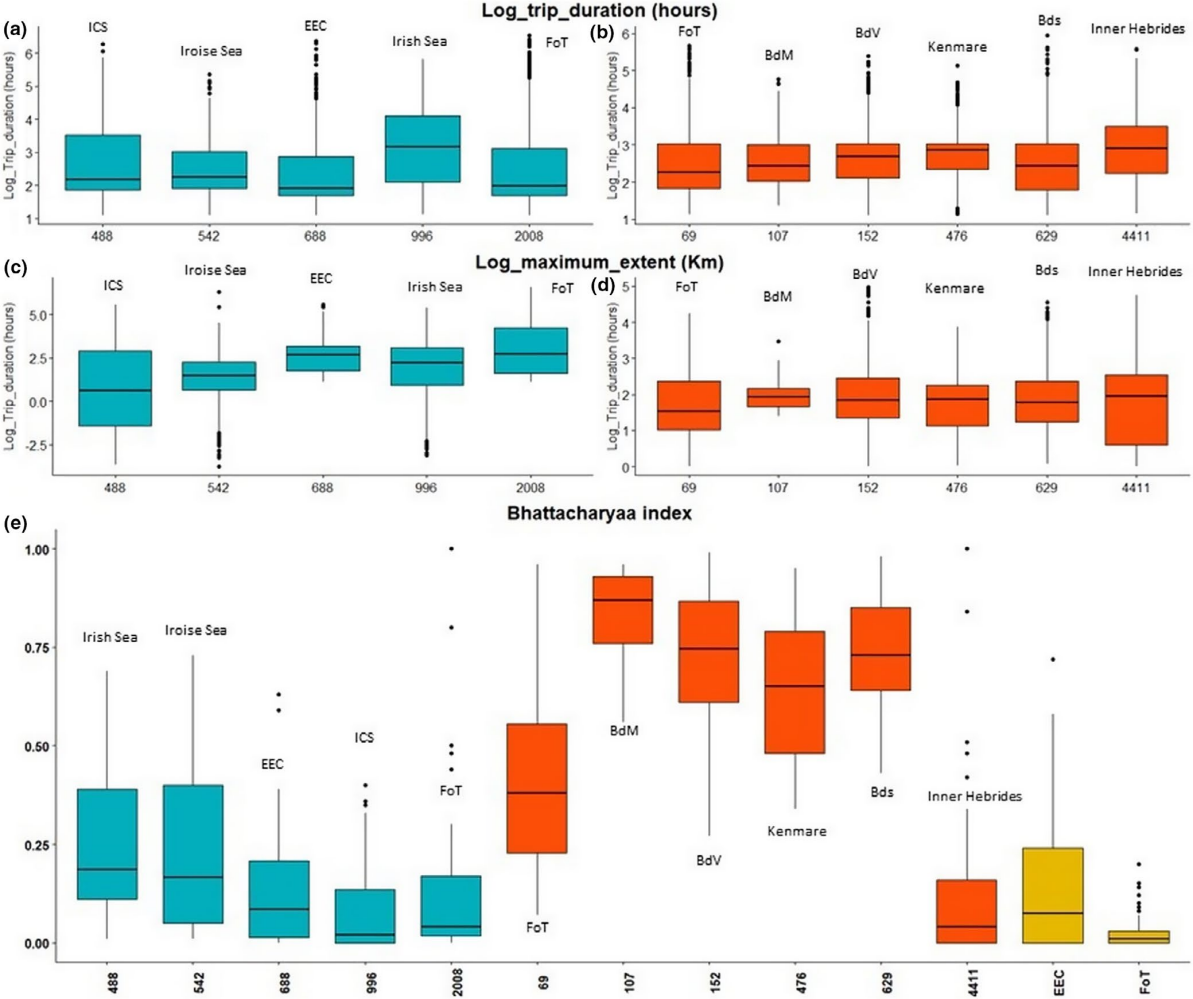
Boxplots of trip characteristics: gray seal (a) and harbor seal (b) trip duration; gray seal (c) and harbor seal (d) maximum extent; and boxplots of Bhattacharyaa index (e); abscise axis represents the number of seals at the colony. Boxplot in blue represents gray seals; in red: harbor seals; in gold: spatial overlap between both species. BdM, Baie du Mont Saint Michel; BdV, Baie des Veys; BdS, Baie de Somme; FoT, Firth of Tay; ICS, Irish Continental Shelf; EEC, Eastern English Channel (including BdS for gray and harbor seals spatial overlap

Means of trip durations were significantly different between sites (*p* < .05, Figure 3, Appendix S4). Most of the pairwise‐site comparisons were significantly different (8/10, *p* < .05). Trip durations were higher for the Irish Sea (median = 3.17 hr; IQR = 1.98 hr) and shorter in the EEC (median = 1.92 hr; IQR = 1.17 hr). Means of maximum extents differed among areas (*p* < .001, Appendix S4). All pairwise comparisons were significantly different (*p* < .05) except for the EEC versus FoT (*p* = .09). Maximum extents were longer for the FoT (median = 2.70 Km; IQR = 2.63 Km) and lower for the ICS (median = 0.626 Km; IQR = 4.30 Km).

#### 3.2.2 Harbor seals’ trip characteristics

Means of trip durations were significantly different between study areas (*p* < .001, Figure 3, Appendix S5). Most of the pairwise‐site comparisons were significant. Trip durations were higher for the Inner Hebrides (median = 2.90 hr; IQR = 1.29 hr), where the individual range was also high and lower for the FoT (median = 2.26 hr; IQR = 1.19). Means of maximum extent differed among sites (*p* < .05). Ten pair‐sites over 17 were significantly different (Figure 3, Appendix S5). Trip maximum extents were higher for the Inner Hebrides (median = 1.94 Km; IQR = 1.94 Km) and lower in the Firth of Tay (median = 1.53 Km; IQR = 1.37 Km).

#### 3.2.3 Measure of similarity in home ranges

Within each site, gray seals individually segregated their spatial usage, indicated by a relative low BA values varying from 0.02 ± 0.12 (Irish Sea) to 0.18 ± 0.18 (ICS) (Figure 3). Overlaps of individual spatial usage were highlighted for harbor seals in the BdM (0.87 ± 0.12), BdV (0.75 ± 0.19), BdS (0.73 ± 0.15), and the Kenmare Bay (0.65 ± 0.19). Conversely, a low BA value was observed for the Inner Hebrides (0.04 ± 0.22). The interspecific comparison between gray seals and harbor seals showed low median value for the FoT (0.01 ± 0.05) and the EEC (0.09 ± 0.17), indicating spatial segregation.

## 4 DISCUSSION

This study highlights the importance of local‐scale effects in understanding the relationship between animals and their environment, particularly in the case of metapopulations where local population trends and physical habitat features vary regionally. By incorporating tracking data from multiple colonies in the Northeast Atlantic (including core population and limit range), our study provides new knowledges on the foraging habitat selection and spatial distribution of gray and harbor seals.

One hundred and two individuals of both species were tracked by telemetry from different colonies from the limit of their range (France and Ireland) to their core population (Scotland), thus depicting habitat selection similarities and differences at the metapopulation level. This represents one of the most extensive datasets available for these species and covers colonies in the core and distributional limits. This compares favorably with habitat selection and spatial usage studies in seabird species (Wakefield et al., 2017) despite a lack of dedicated program for telemetric monitoring of seals at the metapopulation level in the North‐East Atlantic. Previous studies on the ecology of marine mammals, and more specifically pinnipeds, at a comparatively large scale were also based on a compilation of several datasets from different sites to identify habitat use at a global scale (e.g., Baylis et al., 2018). We recognize that the number of individuals tracked for this study may be low to fully characterize the foraging habitat at the scale of colonies. However, our results provide first interesting results on variations of habitat selection between different sites. The choice of the scale of habitat selection is often defined as a mix between the area considered by biologist and managers, as well as the ecology of the species studied. To consider the differences in habitat availability between the different study areas for each species and make inter‐site comparisons, we fitted several models at the local scale. To date, studies encompassing multiple sites with contrasting local habitats have used global models (e.g., Wakefield et al., 2017). We also fitted a global model for each species as it can give more ecological inferences, as here, the influence of colony in the foraging habitat selection. However, although most models’ predictions of this approach were similar to the ones of models at local scale, some ecological inconsistencies were highlighted. For example, the positive influence of bathymetry for harbor seal colonies in the English Channel, which is in contradiction with their ecology in this area as they remain very coastal. Furthermore, the uncertainty obtained around the predictions (i.e., confidence intervals) for the global model, in particular for gray seals, is too important for one part of environmental characteristics. This indicates that the uncertainty in the predictions is too large and might have an impact on the relevance of the results. These differences in predictions suggest problem of homogenization. This was also found by Paton and Matthiopoulos (2016), highlighting the limitations of global models when studying populations that respond to factors at the local scale. That is why, we chose to create a model for each haulout group in order to consider local habitat availability and difference of intraspecific interactions, to avoid this problem of homogenization. We did not include the distribution of seals’ prey resources in the model, as these data were not available for most of the study areas. However, as gray and harbor seals are generalist benthic feeders, we used environmental characteristics that best matched the ecology of their potential prey.

Across models, distance from the last haulout was consistently the most important factor influencing the foraging habitat selection of gray seals in all study areas (from 45% to 76% of the explained deviance in the model respectively for the FoT and the Iroise Sea). These results are consistent with previous studies throughout the range of gray seals in the Northeast Atlantic. Gray seals in the North Sea spent 43% of their time within 10Km of the haulout sites (McConnell et al., 1999) and preferentially selected habitat closer to haulout sites with a gradual decrease of habitat selection beyond tens of kilometers (Aarts et al., 2008). In the Baltic Sea (Sjöberg & Ball, 2000), noted short distance trips (from 10 to 15 Km), gray seals spending 75% of their time within a radius smaller than 50 Km around their haulout sites. Foraging habitat selection by gray seals was negatively influenced by bathymetry, but to a lesser extent than distance from haulout sites (varying from 10% to 40% in the explained deviance). Depending on study sites, the depth selection decreased until a depth of 50 m (Irish and Iroise Sea) and 100 m (FoT). These results are also consistent with previous studies noting usual dive depth between 10 to 80 m (Aarts et al., 2008; Tollit et al., 1998). Gray seals are generally considered as benthic feeders (Beck et al., 2003; Lydersen et al., 1994) and the influence of bathymetry on foraging habitat selection will presumably vary locally depending on the seabed topography and sediment type. In addition to the topography and depth accessibility, seals will search for seabed types favorable to their preys. Furthermore, distance from the shore and tidal current must also influence the behavior of seals, as they use them to orientate, to move, and to forage (Zamon, 2001, 2003). However, these two variables did not contribute much to the explained deviance of the models, and their influence varied among sites. Distribution of seals’ prey resources was not included in the model, as the data were not available for some of the study areas. Results in gray seal spatial usage were in accordance with their habitat selection at the colony scale. There was a global concordance between the foraging habitats selected by gray seals in each site and their spatial pattern, as the prey found in their diet would also be found in the same habitat characteristics (Alheit & Hagen, 1997; Gosch et al., 2014; Hammond et al., 1994; Ridoux et al., 2007). Gray seals in the Firth of Tay and in the Eastern English Channel made longer trips but of shorter duration compared to gray seals in the other sites. In the North Sea, gray seals tend to travel long distances directly to offshore areas on specific sandbanks where sand eel availability is high (Hammond et al., 1994; McConnell et al., 1999; Wilson & Hammond, 2019). This offshore behavior is in accordance with our results for the FoT. Gray seals in the EEC performed their trips mostly in specific areas along the coast, as the EEC is known to be a major ground for flatfish (Carpentier et al., 2009; Riou et al., 2001; Selleslagh et al., 2009), which are observed in gray seal diet (Planque et al., 2021). In the ICS and Iroise Sea, gray seals made shorter trips. These two sites are known as highly biologically productive regions in the Eastern North Atlantic (upwelling and area enclosed by specific currents, respectively, for the ICS and the Iroise Sea (Hily & Glémarec, 1999; Raine & McMahon, 1998). In the Iroise Sea, gray seals selected their foraging habitat around their haulout sites, in the kelp forest. In this area, they are known to forage on wrasse (Ridoux et al., 2007) found on rocky sediment around their haulout sites. However, sediment selected by seals with foraging habitat modeling did not match with rocky substrate. This could be explained by the fact that a selection was made on dives deeper than 3 m, thus excluding those in very shallow waters around their haulout sites (Huon et al., 2015; Vincent et al., 2016). Gray seals in the ICS are located in open oceanic waters, foraging on bentho‐pelagic fish such as sand eel (Ammodytidae), but also consuming a range of pelagic prey including blue whiting (*Micromesistius poutassou*), horse mackerel (*Trachurus trachurus*), silvery pout (*Gadiculus argenteus*), and garfish (*Belone belone*) in contrast to the Irish Sea where diet is mainly composed by ray (Rajidae), dragonnets (*Callionymus* spp., Callionymidae) and soles (Soleidae) (Gosch et al., 2019), which can be found over muddy and sandy area in shallow waters (Ellis et al., 2000), corresponding to habitat selected by gray seals in this area.

Distance from the last haulout, distance from shore, and/or bathymetry explained most of the deviance (>90%) in harbor seal foraging habitat selection. These three variables had a negative influence, but at different degrees depending on the site configuration. Harbor seals were very coastal and sedentary in the six study areas, which was also supported by their spatial patterns. This is consistent with previous findings on the species; in the Moray Firth (East of Scotland) for instance, seals forage within 30 Km of their haulout sites and dive at a maximum depth of 50 m (Bailey et al., 2014; Tollit et al., 1998). This was also highlighted on the other side of the Atlantic, in the Saint Laurent estuary (Lesage et al., 2004), seals were coastal (with distances shorter than 11 Km from the shore) and in shallow waters (<50 m deep). Tidal current and sediment types accounted less in the explained deviance than the other variables. Their influence was generally very low, with the exception of the tidal current for the FoT. Harbor seals feed on diverse fish species and their diet vary locally (Hall et al., 1998). At their southern limit range, in the English Channel, harbor seals mainly selected foraging habitat over mixed sediments in coastal areas and estuaries, that is, where there are nurseries of flatfish species, and that is concordant with harbor seal diet in the BdS essentially including small benthic flatfish (Spitz et al., 2015). Seal diet in BdM was not available so we could not compare seals’ foraging habitat selection and known prey habitat in this region. However, on the East coast of Scotland, harbor seals selected habitat over sandy areas in front of the Tay river mouth, corresponding to the habitat of sandeel—the main harbor seal prey in the area (Wilson & Hammond, 2019). Harbor seals in Inner Hebrides essentially forage on demersal fish (e.g., whitings *Merlangius merlangus*) and pelagic fish (e.g., herrings *Clupea harengus*) (Wilson & Hammond, 2019); therefore, their benthic component may be less important than in other sites and that could be concordant with their habitat selection along the sounds of the fjords. Sediments and current features selected by harbor seals might correspond to the habitat features of their prey.

Distance from the last haulout was the most important factor influencing habitat selection for both species and all colonies. This is in accordance with the theory of central place foraging where animals will minimize distance‐dependent travel costs (Orians & Pearson, 1979) and shows the importance of including not only potential quality of habitat, but also elements related to species biology. This has been demonstrated in many central place foraging species, both herbivores (Gerwing et al., 2013; Shrader et al., 2012) and carnivores (Rainho & Palmeirim, 2011). Results of habitat selection and spatial usage for both species highlighted the importance of considering local‐scale effects within a metapopulation. Indeed, despite the fact that the colonies are a few hundred kilometers apart, the colonies were located in contrasting environments with different habitat characteristics and associated prey availability. The predator–prey relationship varies spatially in association to the underlying physical conditions (Santora et al., 2014). Gray seal and harbor seal are two species with a high behavioral plasticity and the importance of behavioral plasticity in environmental adaptation has been ably demonstrated for multiple species, as for example in squirrels (Hefty & Stewart, 2019). All animals experience dynamic physiological and environmental demands and must adjust their activity patterns to minimize associated cost and increases fitness. Due to differences in habitat availability and habitat selection among colonies, our results on spatial usage did not highlight any potential effect of density dependence on foraging ranges. Various factors other than density dependence can also impact spatial patterns. Animals should adjust their foraging movements for habitat exploitation, depending on environmental heterogeneity and hierarchical distribution of resources (Pinaud & Weimerskirch, 2007). This was already demonstrated in seabird species, where the influence of the shape of the coast and the availability of habitat further away from the central place forces birds to forage further offshore without there necessarily being a density effect (Wakefield et al., 2017). In our study, this may have been the case of gray seals in the FoT foraging on sandbanks in the middle of the North Sea. Phenotypic plasticity was also highlighted to influence spatial usage for fur seals (Baylis et al., 2018).

The number of harbor seals has declined at some colonies in the North Sea during the last decade (Thompson et al., 2019). The decline of sandeel numbers (main prey of harbor seals around these colonies) and interspecific competition with gray seals were suggested as one of the potential causes in local harbor seal declines (Wilson & Hammond, 2019). In our study, gray seals and harbor seals were tracked in two areas where both species haul‐out (FoT and EEC), and spatial partitioning between seal species was highlighted in habitat selection and spatial patterns in these areas. Gray seals made longer trips than harbor seals (15 Km for median maximum extents in gray seals in both areas, versus 5 Km and 4 Km respectively for harbor seals in the EEC and FoT). In both cases, harbor seals tended to forage in inshore areas, while at least some of the gray seals foraged further offshore, which is consistent with previous studies that also found differences in the use of marine environment between these two species (Jones et al., 2015; Sharples et al., 2012). However, Planque et al. (2021) found trophic overlap between both species in the EEC.

In the North Atlantic, gray seals and harbor seals are managed at local scales, and in the absence of genetic information on population structuring, haulout groups are often considered as “Seal Management Units” (Russell et al., 2013). Both species are considered as generalist, using a variety of habitats and prey. This study highlights the importance of studying foraging habitat selection at local scale and considering the variability between colonies, as physical habitat features and seals’ prey resources vary between regions. As marine top predators, both seal species are listed in the Annex II of the European habitat directive requiring establishment of protected areas to maintain favorable conservation status. At a local scale, our predictive maps of foraging habitat selection could be used by managers to implement specific areas of conservation to maintain a good ecological state of their habitat and prey resources potentially at risk due to anthropic activities. Such a foraging habitat selection analysis could be applied and/or adapted for other central place foraging species, in both marine and terrestrial ecosystems.

## CONFLICT OF INTEREST

The authors have no conflict of interest to declare.

## AUTHOR CONTRIBUTIONS

**Mathilde Huon:** Conceptualization (lead); Formal analysis (lead); Funding acquisition (equal); Investigation (lead); Methodology (lead); Supervision (equal); Validation (lead); Writing‐original draft (lead); Writing‐review & editing (lead). **Yann Planque:** Methodology (equal); Validation (equal); Writing‐original draft (supporting); Writing‐review & editing (supporting). **Mark John Jessopp:** Data curation (equal); Validation (equal); Writing‐original draft (supporting); Writing‐review & editing (supporting). **Michelle Cronin:** Data curation (equal); Validation (supporting); Writing‐original draft (supporting); Writing‐review & editing (supporting). **Florence Caurant:** Conceptualization (supporting); Project administration (supporting); Supervision (equal); Validation (supporting); Writing‐original draft (supporting); Writing‐review & editing (supporting). **Cécile Vincent:** Conceptualization (supporting); Data curation (lead); Funding acquisition (lead); Methodology (supporting); Project administration (equal); Supervision (equal); Validation (equal); Writing‐original draft (supporting); Writing‐review & editing (supporting).

## Supporting information

Supplementary Material

Supplementary Material

Supplementary Material

Supplementary Material

Supplementary Material

Supplementary Material

Supplementary Material

## Data Availability

The data that support the findings of this study are openly available in SEANOE at https://doi.org/10.17882/81072 (Irish dataset), https://doi.org/10.17882/81079 (Scottish dataset) and https://doi.org/10.17882/81033 (French dataset).

## ENDNOTES

^1^

http://www.smru.st‐and.ac.uk/Instrumentation/downloads/GPS_Phone_Tag22.pdf

^2^
http://www.emodnet.eu/bathymetry,

^3^

https://www.marine.ie/Home/home

^4^
*ABP mer*, http://renewables‐atlas.info/
