# Supplementary figures and images for: Fine‐scale foraging habitat selection by two diving central place foragers in the Northeast Atlantic
Source: Ecol Evol. 2021 Aug 24;11(18):12349–63. doi: 10.1002/ece3.7934 (PMC8462179; doi:10.1002/ece3.7934)

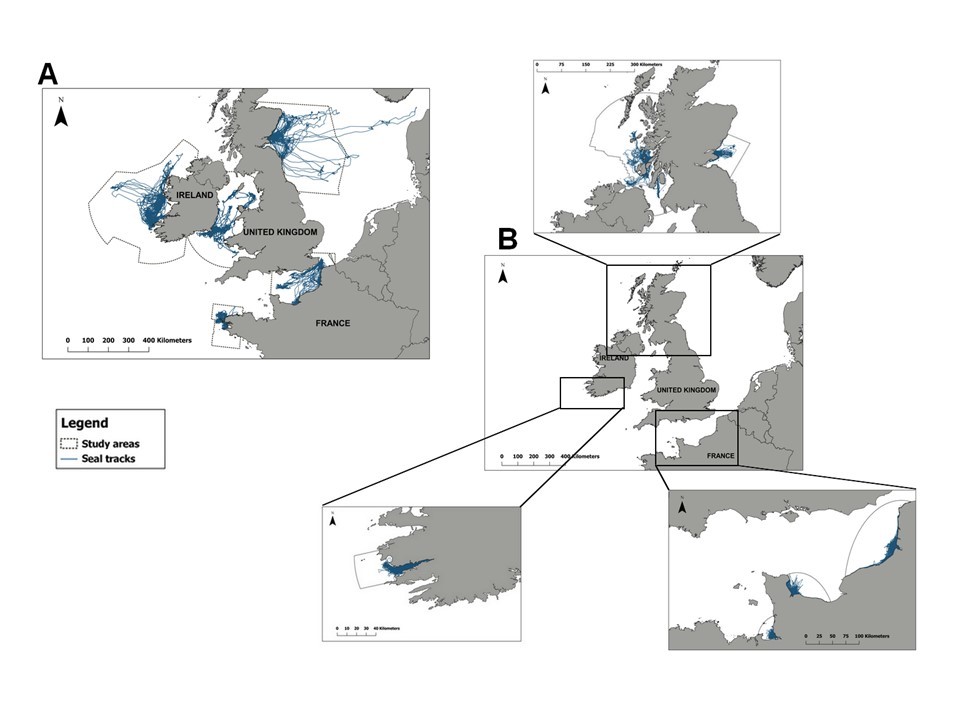

Supplement: Supplementary file 1 — Supplementary Material [file ECE3-11-12349-s007.jpg]

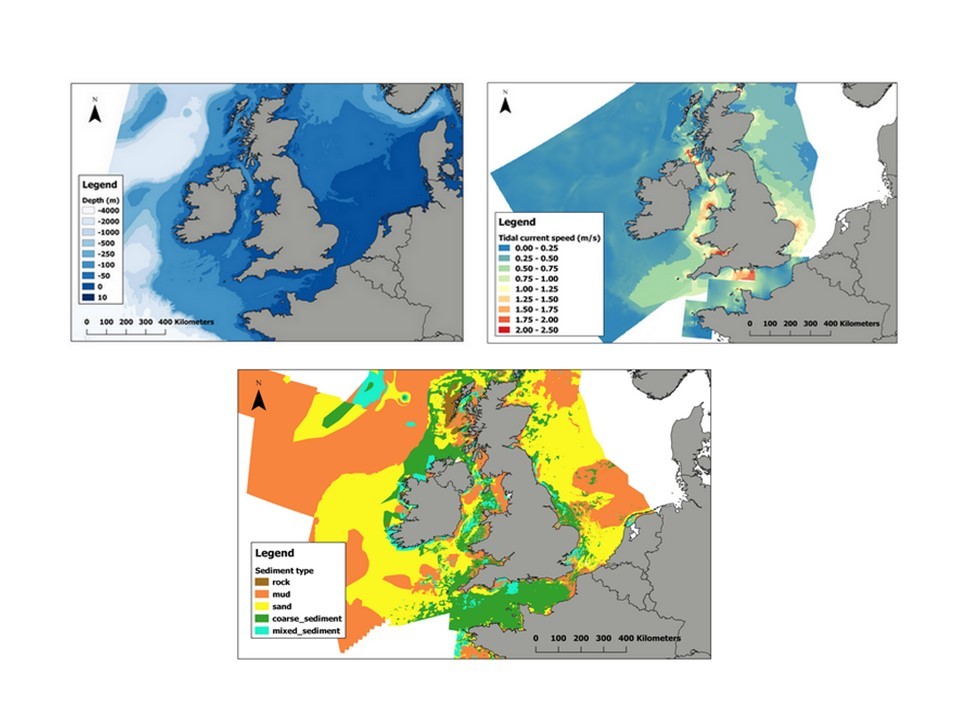

Supplement: Supplementary file 2 — Supplementary Material [file ECE3-11-12349-s004.JPG]

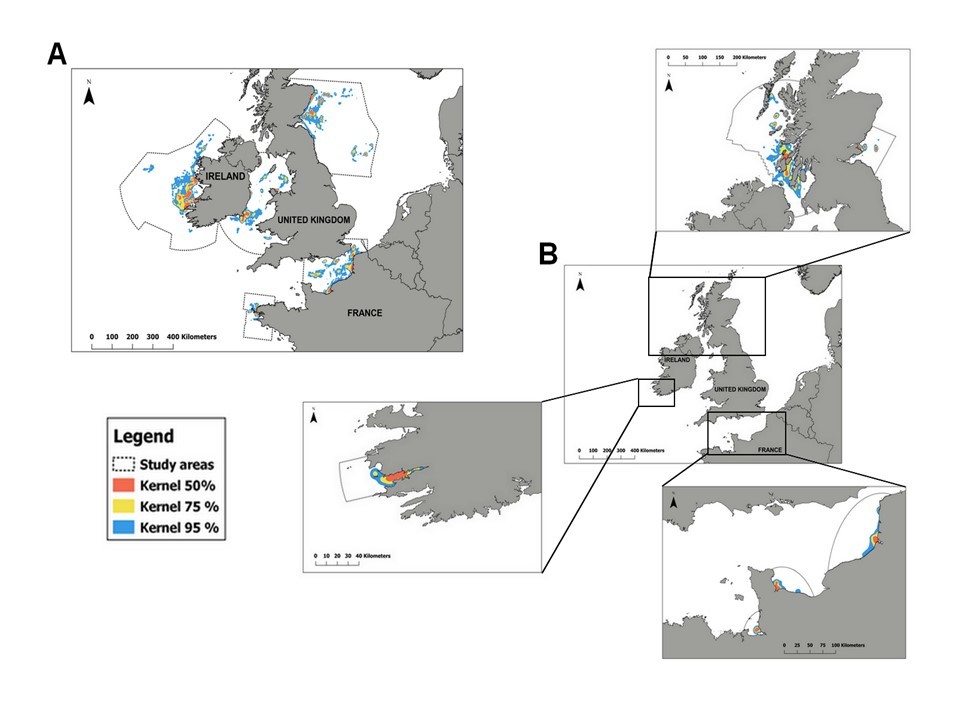

Supplement: Supplementary file 3 — Supplementary Material [file ECE3-11-12349-s005.jpg]

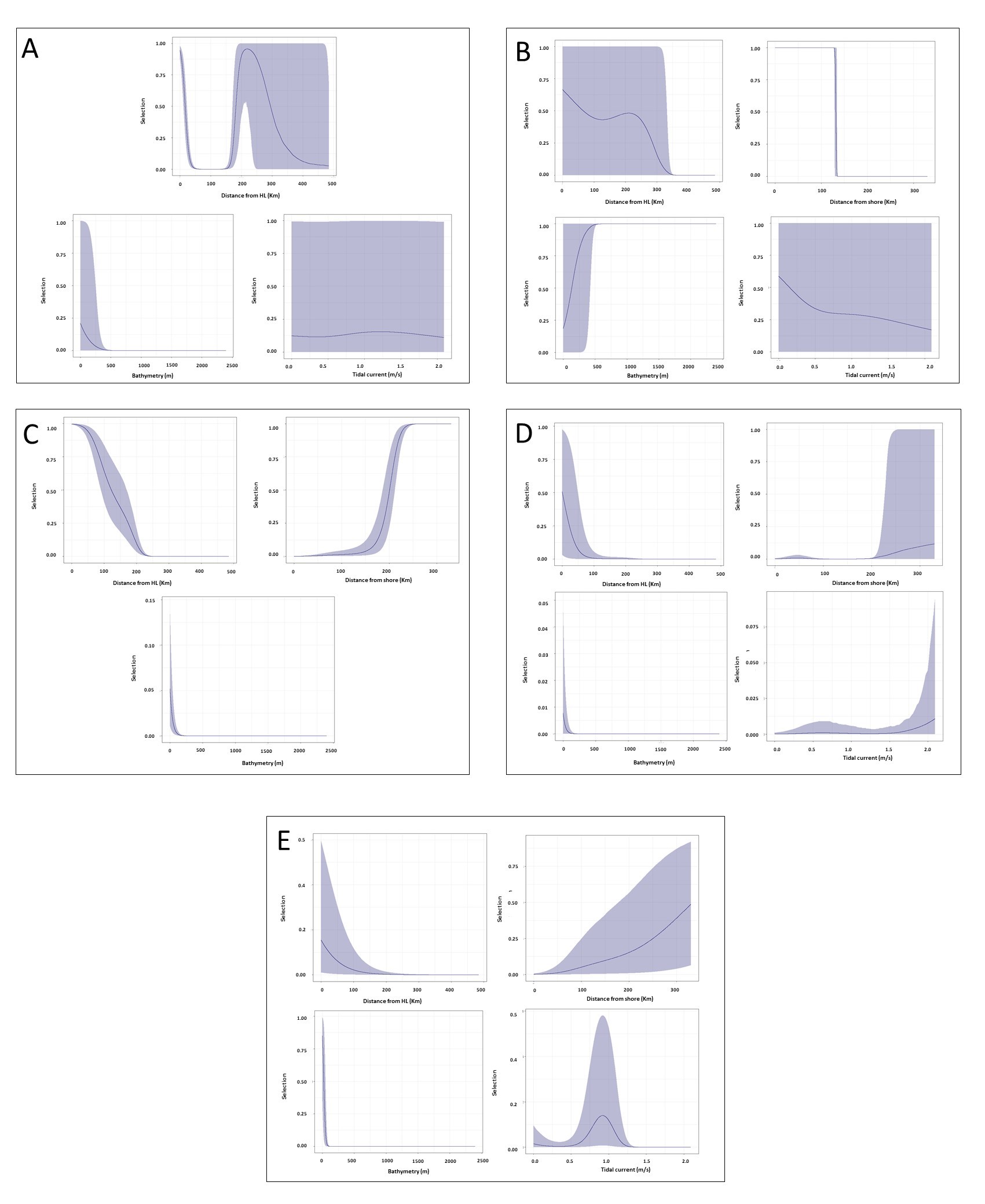

Supplement: Supplementary file 4 — Supplementary Material [file ECE3-11-12349-s003.jpg]

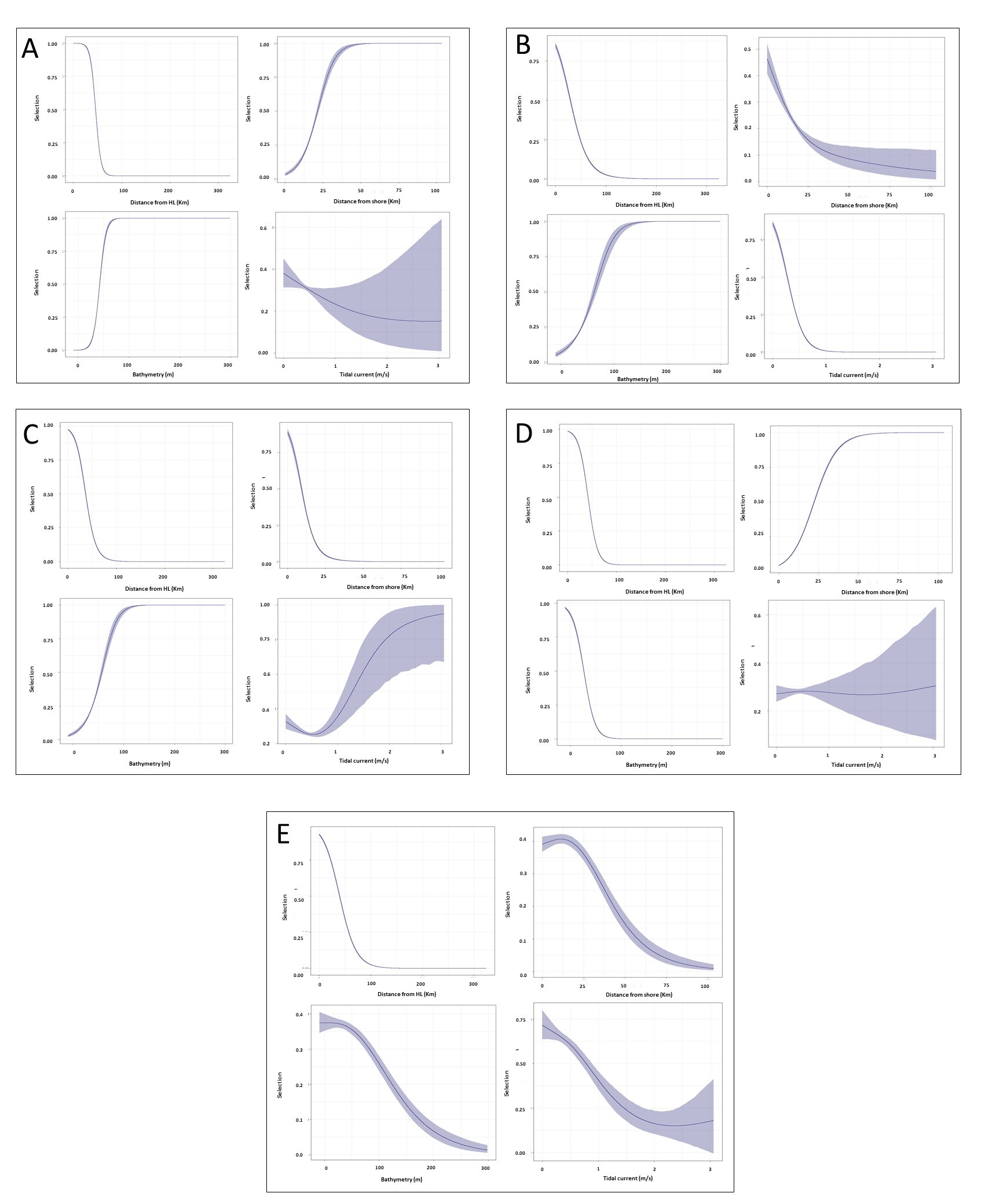

Supplement: Supplementary file 5 — Supplementary Material [file ECE3-11-12349-s002.jpg]

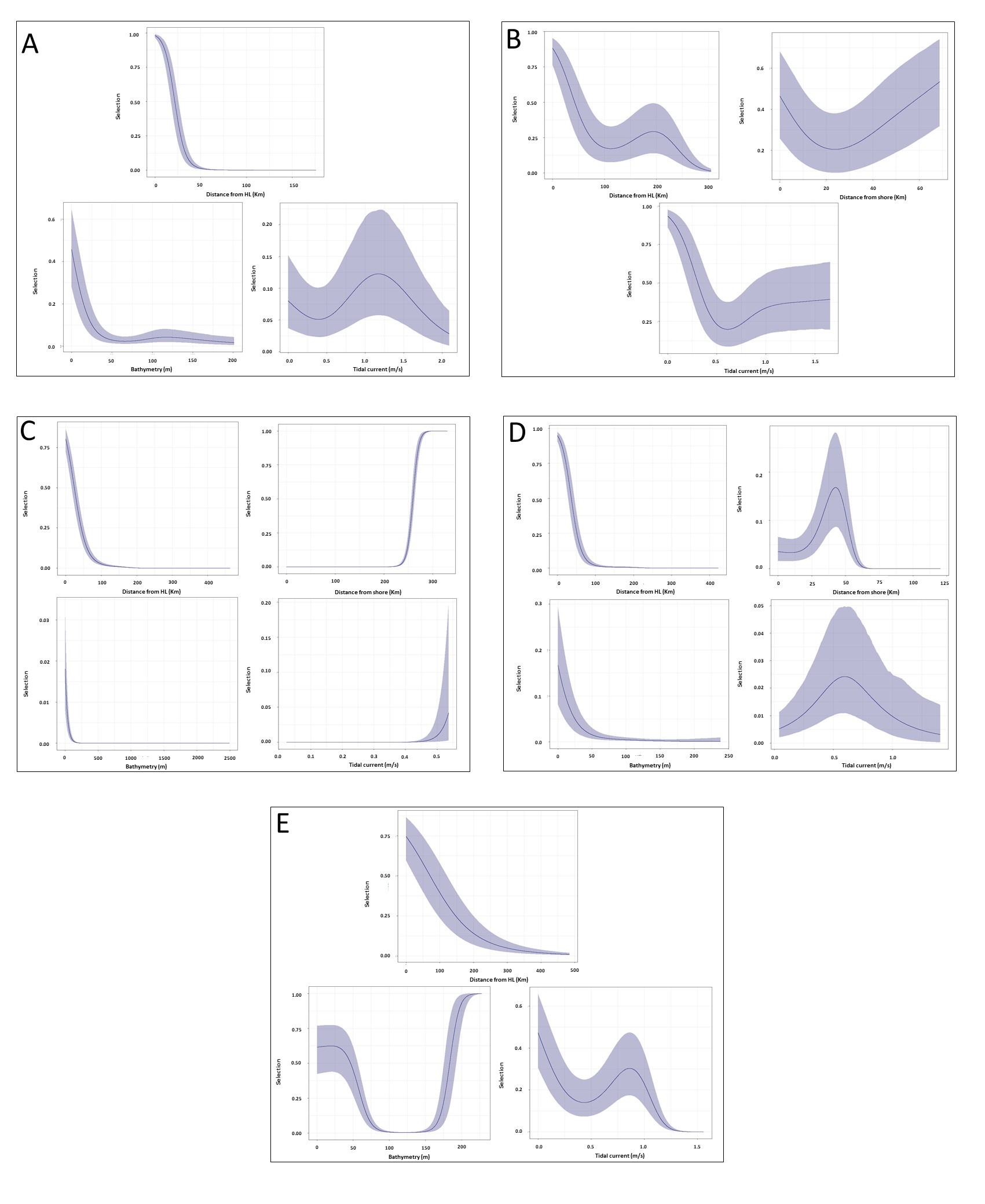

Supplement: Supplementary file 6 — Supplementary Material [file ECE3-11-12349-s006.jpg]

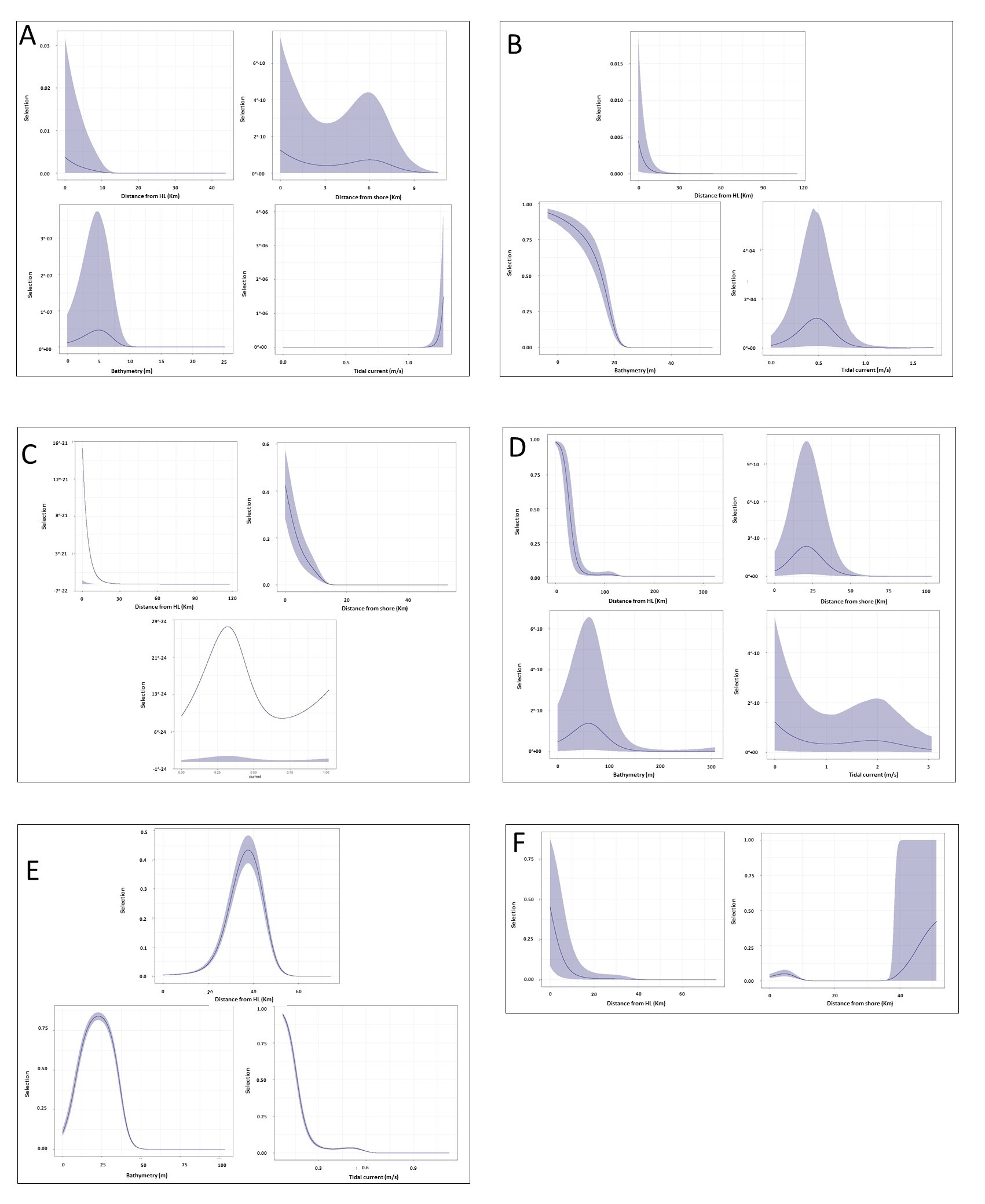

Supplement: Supplementary file 7 — Supplementary Material [file ECE3-11-12349-s001.jpg]
